# The Effect of Short-Term Annealing of the Amorphous Metal Alloy Al_87_Y_4_Gd_1_Ni_8_ on Surface Morphology and Electrochemical Properties

**DOI:** 10.3390/ma19040670

**Published:** 2026-02-10

**Authors:** Khrystyna Khrushchyk, Julian Kubisztal, Katarzyna Balin, Krzysztof Aniołek, Vasyl Kordan, Małgorzata Karolus, Lidiya Boichyshyn

**Affiliations:** 1 Faculty of Chemistry, Department of Physical and Colloidal Chemistry, Ivan Franko National University of Lviv, 6 Kyryla and Mefodiia Str., 79005 Lviv, Ukraine; khrystyna.khrushchyk@us.edu.pl (K.K.); lidiya.boichyshyn@lnu.edu.ua (L.B.); 2 Faculty of Science and Technology, Institute of Materials Engineering, University of Silesia in Katowice, 1A Pulku Piechoty Str., 41500 Chorzow, Poland; julian.kubisztal@us.edu.pl (J.K.); katarzyna.balin@us.edu.pl (K.B.); krzysztof.aniolek@us.edu.pl (K.A.); 3 Faculty of Chemistry, Department of Inorganic Chemistry, Ivan Franko National University of Lviv, 6 Kyryla and Mefodiia Str., 79005 Lviv, Ukraine; vasyl.kordan@lnu.edu.ua

**Keywords:** amorphous metal alloy (AMA), short-term annealing, corrosion, oxide layer, roughness

## Abstract

**Highlights:**

**What are the main findings?**

- Short-term annealing increases surface roughness and active area, while prolonged annealing induces partial surface leveling.
- Corrosion resistance of amorphous Al_87_Y_4_Gd_1_Ni_8_ in 0.3% NaCl is strongly dependent on annealing duration.

**What are the implications of the main findings?**

- Enhanced surface activity after short-term annealing may reduce protective performance.
- Prolonged annealing decreases film protectiveness despite surface smoothing.

**Abstract:**

Amorphous metal alloys (AMAs) are characterized by good mechanical and electrochemical properties. However, due to crystallization processes occurring at higher temperatures (Ta ˃ 600 K), these properties may deteriorate. The aim of this work was to investigate the effects of short-term annealing at T_3_ = 611 ± 1 K and to determine the risks of such thermal modifications for the electrochemical properties of the material. A comprehensive analysis shows that short-term isothermal annealing (5 min) of the amorphous metal alloy Al_87_Y_4_Gd_1_Ni_8_ at a temperature of 611 ± 1 K improves the tribological properties of the material. However, it has been established that heat treatment for 5 min is optimal and leads to temporary thickening of the film and the formation of an almost ideal double layer, but annealing for 15–60 min negatively affects the electrochemical properties of this material, indicating a decrease in the protective properties of the passivating layers.

## 1. Introduction

Aluminum alloys are increasingly used in industry, being widely used in aerospace and automotive applications due to their good mechanical properties [1,2,3]. Aluminum-based AMA heat treatment is used to improve corrosion resistance or wear resistance, as well as to create special electrical, optical, hydrophilic or adhesive properties [4,5]. Thermal annealing changes the surface of the amorphous alloy, which affects the electrochemical properties of the material. A passive film can be defined as a complex membrane structure consisting of various insoluble oxide layers that protect materials from corrosion [6]. In some cases, the pitting resistance of an alloy is determined by the passive film formed on its surface. It is known that there are two main ways to increase pitting resistance: reducing the migration of anions into passive films [7,8] and increasing the critical pitting current density [9,10].

The articles present the results of studies on the corrosion resistance of the main groups of Al-based [11,12,13,14], Fe-based [15,16,17,18,19,20,21,22,23], Co-based [24], Ni-based [25,26] and Mg-based [27,28] amorphous metal alloys. The authors of article [29] showed that depending on the morphology, oxide layers are also divided into non-porous oxide layers of the barrier type and porous oxide layers. Depending on the electrolyte, non-porous barrier layers can be formed in neutral electrolytes (pH 5−7) of borate phosphate, adipate, etc., solutions. Porous ones are formed in acidic electrolytes of selenium, sulfuric, oxalic, phosphoric, chromic, malonic, tartaric, citric and malic acids [29,30,31,32].

The presence of Cl ions accelerates the release of metal into solution [33], which can disperse the oxide film to a colloidal level of dispersion, thereby increasing its permeability [33]. It is known that the first stage of pitting is the adsorption of chloride ions on the oxide-coated surface. When an ion interacts with an ionic surface such as oxide, the attractive forces consist of: (1) Coulomb forces; (2) induction of the adsorbent by a neighboring ion; (3) electrostatic polarization of the ion; and (4) non-polar Van der Waals forces. Of these, the first two interactions of an ionic nature make the largest contribution. In neutral solutions, the oxide film on aluminum is formed by hydration:

(1) $$\mathrm{Al}(\mathrm{oxide})\;\mathrm{OH}\;+\;H^{+}\;\to \;\mathrm{Al}(\mathrm{oxide})\;{OH}_{2}^{+}$$

(2) $$\mathrm{Al}(\mathrm{oxide})\;{OH}_{2}^{+}+{\mathrm{nCl}}^{-}\;\to \;\mathrm{Al}(\mathrm{oxide})\;{OH}_{2}^{+}{Cl}_{n}^{-n}$$

The second stage of pitting corrosion is the penetration of chlorine ions into the oxide film on aluminum, the exact mechanism of which is unknown. It has been suggested that the corresponding effect is Al(oxide) ${OH}_{2}^{+}{Cl}_{n}^{-n}$ + nV_0··_ → Al[n(Cl_0·_)(oxide) ${OH}_{2}^{+}$.

Further dissolution of Al, which occurs under the oxide film at the metal/oxide interface, can be described by the following equations:

(3) $$\mathrm{Al}[n({\mathrm{Cl}}_{0\cdot })(\mathrm{oxide})]\;{OH}_{2}^{+}\to \;{\mathrm{Al}}^{+}\;[n({\mathrm{Cl}}_{0\cdot })(\mathrm{oxide})]\;{OH}_{2}^{+}\;+\;e^{-}$$

(4) $${\mathrm{Al}}^{+}\;[n({\mathrm{Cl}}_{0\cdot })(\mathrm{oxide})]\;{OH}_{2}^{+}\;\to \;{\mathrm{Al}}^{++}\;[n({\mathrm{Cl}}_{0\cdot })(\mathrm{oxide})]\;{OH}_{2}^{+}\;+\;e^{-}$$

In Al(oxide)OH, Al corresponds to the atom of the metallic aluminum substrate located directly under the oxide film; (oxide) means the oxide film covering the aluminum substrate, which undergoes local dissolution (pitting); OH is the outer layer of surface hydroxyl groups. Additionally, V_0··_ signifies oxygen vacancies in the film; Cl_0·_ signifies a chloride anion that has taken the place of oxygen in the structure; Al(oxide) ${OH}_{2}^{+}$ is the oxide film on aluminum through which chlorine ions are transferred; Al(пCl_0·_)(oxide) ${OH}_{2}^{+}$ is aluminum with zero valence, which exists before pitting, covered with an oxide film containing Cl^−^ ions; and Al^+^[(пCl_0·_)(oxide)] ${OH}_{2}^{+}$, Al^++^ [n(Cl_0·_)(oxide)] ${OH}_{2}^{+}$ and Al^++^ [n(Cl_0·_)(oxide)] ${OH}_{2}^{+}$–, respectively, are one-, two- and three-valent aluminum ions at the metal/oxide interface, i.e., at the sites of pitting initiation [34]. The authors of article [35] divided the immersion process of an amorphous aluminum-based alloy into five zones: (i) The first zone involved the growth of a passive film. (ii) In the second zone, a complete passive film formed, and a dynamic and stable process took place. A passive film occurred in the third zone. (iii) In the fourth zone, corrosion products accumulated on the surface of the amorphous aluminum-based alloy, and a layer of corrosion products was formed. (iv) In the fifth zone, the diffusion of corrosion products contributed to the formation of continuous corrosion channels.

The change in the morphology of the amorphous alloy after annealing significantly affects the electrochemical properties of the alloy. The authors of [36] described the corrosion properties of Al_84_Ni_9_Y_7_ alloy samples in a 3.5% NaCl solution after different annealing conditions. According to the results of the studies, Al_2_O_3_, NiO, Y_2_O_3_ and Al(OH)_3_ compounds were identified in the surface layer. The composition of the protective layer was studied in more detail by the authors of [37], who used the XPS method in their work. If, after heat treatment of the Al_86_Ni_10_Y_4_ alloy, O 1s, Al 2p, Ni 2p3/2 and Y 3d5/2 signals were observed in the spectrum of the relaxed sample, then in the spectrum of the sample annealed at 400 °C, Ni 2p3/2 reflections were not detected, which indicates a significant enrichment of the Y surface after annealing. It is well known that yttrium is a promoter of passivity, and Y_2_O_3_ has high thermodynamic stability in aqueous environments [38]. According to the Purbe diagram [38], yttrium forms a stable passive film in the pH range of 6.5–16.0, i.e., as expected, the annealed sample, due to the increase in Y content in the surface layer, forms a stable oxide–hydroxide film in the test solution, the pH of which varies from 6.5 to 7.3 during the experiments. The decisive factor that can be used to regulate the corrosion resistance of aluminum-based AMAs is the duration and temperature of annealing.

The authors of article [39] concluded the following: (1) At temperatures below 300 °C, the oxide film formed on the surface of pure aluminum is an amorphous aluminum oxide several nanometers thick [40]. (2) Amorphous aluminum oxide begins to transform into γ-Al_2_O_3_ at temperatures above 300 °C. The surface oxide film of aluminum foil transforms from amorphous aluminum oxide to crystalline γ-Al_2_O_3_ at temperatures above 450 °C [41]. (3) The amorphous oxide film on the surface of aluminum foil transforms into crystalline γ-Al_2_O_3_ at 680 °C [42]. Wang et al. [43] analyzed the oxide film formed on the surface of molten aluminum at 750 °C and concluded that the oxide film is crystalline γ-Al_2_O_3_. The authors of article [13] show that structural relaxation annealing (annealing temperatures lower than the nanocrystallization onset temperature) significantly increases the corrosion resistance of Al-Ni-Y alloys in a 0.6 M NaCl solution due to a reduction in the free volume of the samples, which promotes the formation of a highly protective passive film. For a sample annealed for 5 min at 150 °C, the value of the passivation current density (iпac) decreases to 1.87−0.12 μA/cm^2^, and the pitting potential (E_pit_) shifts to the range of −89 … +10 mV (SCE), indicating better corrosion resistance.

Thus, thermal modification of AMAs significantly affects their physical and chemical properties, in particular corrosion resistance [37,44]. However, contradictory results have been reported in the literature. In particular, the authors of works [24,25,26] indicate that controlled annealing at temperatures below the crystallization threshold (T_1_) increases the corrosion resistance of metal glasses such as Al–Co–Ce, Cu–Ti, Co–Fe–Mo–Si–B, Fe–B–Si and Fe–B–Si–Cr. However, similar studies of Fe–Cr–P–C [13] and Fe Cu–Nb–Si–B [14] alloys have shown the opposite effect. These results indicate that the corrosion properties of AMAs are quite sensitive to their composition. Corrosion is a typical heterogeneous process, and its rate depends on both the size of the phase boundary area (metal and corrosive medium) and the composition of both phases. Annealing of samples below the crystallization temperature leads to a change in the composition of the AMA surface, as shown in [45], which can also greatly affect the corrosion rate. Therefore, this work is an attempt to carry out an investigation into the effects of isothermal annealing on the morphology of AMAs and the electrochemical properties of alloys at a temperature of constant crystallization of the second peak of structural transformations 611 ± 2 K, with annealing times of 5, 15, 30, 45 and 60 min, and to assess the risks of such thermal annealing for the amorphous metal alloy Al_87_Y_4_Gd_1_Ni_8_.

## 2. Materials

The objects of the investigations were AMAs in the form of ribbons with a thickness and width of 20–25 μm and 3 mm, respectively, which were obtained at the G. V. Kurdyumov Institute for Metal Physics of the Ukrainian Academy of Sciences (Kyiv) by the melt spinning method in a helium atmosphere on a copper drum rotating at a speed of ~30 m/s. The melt was prepared from pure metals and binary compounds REAl_3_ (RE = Y and Gd). The purity of the starting metals was as follows: Al (99.999 wt.%), Ni (99.99 wt.%), Y (99.96 wt.%), Gd (99.96 wt.%) and Fe (99.99 wt.%). This work investigates changes in the morphology and electrochemical properties of an amorphous metal alloy Al_87_Y_4_Gd_1_Ni_8_ in a 0.3% aqueous NaCl solution as a result of annealing. Isothermal annealing (at 5, 15, 30, 45 and 60 min) was performed at certain temperatures with a heating rate 20 K/min determined from DSC curves, which are characteristic of the stable growth of crystals of the secondary crystallization stage and equal to 611 ± 2 K.

## 3. Methods

### 3.1. Morphology Analysis

#### 3.1.1. Surface Roughness

Surface roughness and wear mark geometry tests following tribological testing were performed using a Mitutoyo Surftest SJ-500 2D contact profilometer (Mitutoyo, Tokyo, Japan). Roughness measurements of the samples were performed in the longitudinal direction. Roughness was measured in accordance with ISO 21920 using an elementary section and a measuring section selected according to the surface roughness [46].

#### 3.1.2. Tribological Characteristics

Tribological characteristics were tested using a TRN Tribometer operating in a ball-on-disc system (Anton Paar, Corcelles-Cormondrèche, Switzerland). The tribological tests were performed in a linear reciprocating motion in dry contact. The test parameters were selected on the basis of preliminary tests. The tests were performed at a load of 1 N with a frequency of 5 Hz over a friction distance of 100 m. The amplitude of movement was 10 mm. The maximum linear speed was 15.71 cm/s. The tests were carried out at a temperature of 21 ± 1 °C and a humidity of 40 ± 5%. Balls with a diameter of 6 mm made of zirconium oxide (ZrO_2_) were used as counter-samples. After the tribological tests, the average surface area of the wear marks was evaluated using a Surftest SJ-500 contact profilometer (Mitutoyo, Tokyo, Japan).

#### 3.1.3. XPS Method

The elemental composition of the Al_87_Y_4_Gd_1_Ni_8_ samples was examined via X-ray photoelectron spectroscopy (XPS). The XPS analyses were conducted using a Physical Electronics PHI 5700 spectrometer (Chanhassen, MN, USA), equipped with a monochromatic Al Kα X-ray source (1486.6 eV). Data acquisition was performed on as-prepared surfaces. The analyzed regions, defined by an aperture, had a diameter of 80 μm. Both survey and high-resolution spectra were recorded at a 45° take-off angle, with pass energies of 187.85 eV (step size 0.8 eV) for survey scans and 23.50 eV (step size 0.05 eV) for detailed core-level spectra. To mitigate sample charging caused by X-ray exposure, a flood gun was employed. Quantitative analysis and spectral fitting were carried out using MULTIPAK software (v.9.6.0.1, ULVAC PHI, Chigasaki, Japan). To correct for surface charging, the measured spectra were referenced to the C1s peak at a binding energy of 284.8 eV. Core-level spectra were deconvoluted using a Shirley background subtraction and mixed Gaussian–Lorentzian peak profiles.

#### 3.1.4. Electrochemistry

The change in the electrochemical properties of amorphous metal alloys as a result of AMAs annealing in a 0.3% NaCl aqueous solution at a temperature of 293 K was investigated. The following electrochemical parameters were established: R_s_, R_f_, CPE-f-T, R_ct_, CPE-dl-T and n_dl_. The following methods were used to determine the change in electrochemical parameters: open-circuit potential (OCP method); experimental conditions: working electrode (WE)—amorphous metal alloy with a working area of 0.5 cm^2^; reference electrode (RE)—saturated calomel electrode, SCE, saturated calomel (sat’d KCl) = 0.242 V; the electrical equivalent elements used in the models are solution resistance (Rs), charge transfer resistance (R_ct_) and corrosion capacitance of the medium/corrosion product interface (CPEdl); charge transfer resistance (R_ct_) is an important parameter and is inversely proportional to the corrosion rate for each type of coating [47] (electrochemical impedance spectroscopy, EIS method); initial frequency: 20.0 kHz; final frequency: 10.0 MHz. These experiments were performed using a PARstat 2273 Revision 2273 potentiostat (Princeton Applied Research Ametek, Berwyn, PA, USA).

## 4. Results and Discussion

### 4.1. Profile Analysis

**Figure 1:**
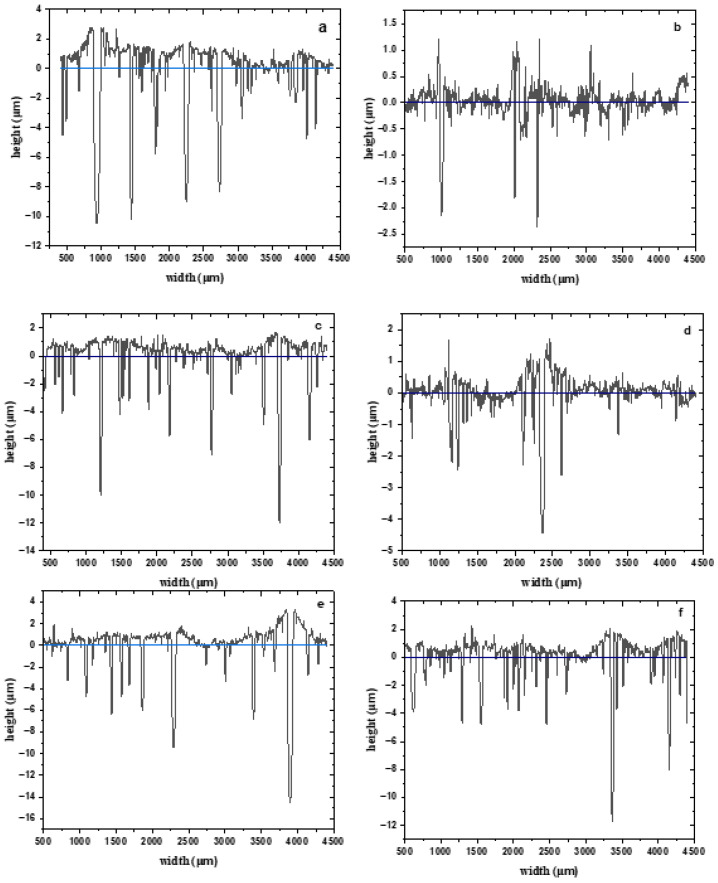
Amorphous metal alloys (AMAs) surface profiles of the contact side (width × height): (**a**) initial; (**b**–**f**) annealed: (**b**) 5 min, (**c**) 15 min, (**d**) 30 min, (**e**) 45 min and (**f**) 60 min.

A profile analysis of the surface (Figure 1) of the amorphous alloy Al_87_Y_4_Gd_1_Ni_8_ showed significant changes in the topography depending on the heat treatment mode. In the as-cast state, the surface is characterized by relatively low roughness and a relatively uniform distribution of protrusions and depressions, which corresponds to a compact protective film. After short-term annealing (5 min), an increase in the average profile height and roughness is observed, indicating the opening of small structural defects and an increase in the active surface area. Longer annealing (15–30 min) leads to the formation of more pronounced irregularities and local depressions, reflecting partial destruction or porosity of the film, and roughness reaches its maximum values. Furthermore, at 45–60 min of heat treatment, the surface profile becomes more uniform, but the roughness decreases, which may be associated with the partial melting of small protrusions and the leveling of the surface topography.

Profile analysis shows that short-term annealing increases the active area and surface roughness, while prolonged annealing leads to partial leveling but with a simultaneous decrease in the protective properties of the film. These results are consistent with electrochemical impedance spectroscopy data, where maximum roughness corresponds to increased CPE and reduced film resistance.

The main roughness parameters were calculated from these profilograms, which are shown in Table 1.

**Figure 2:**
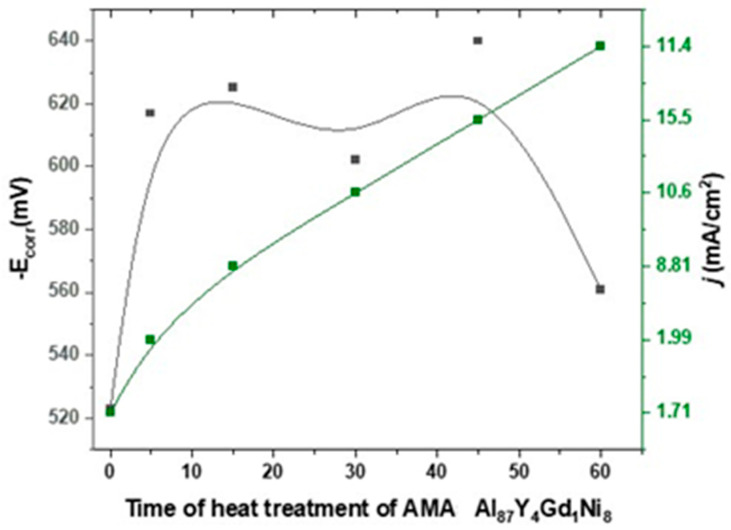
Evolution of the corrosion potential (E_corr,_ black line, left axis) and corrosion current density (*j*, green line, right axis) of the amorphous Al_87_Y_4_Gd_1_Ni_8_ alloy as a function of heat treatment time in 0.3% NaCl solution.

Profile analysis of the surface of the amorphous alloy Al_87_Y_4_Gd_1_Ni_8_ after various heat treatment modes showed significant changes in the topography. In the as-cast state, the surface is characterized by a relatively uniform profile with an average roughness of R_a_ = 2.83 μm, a quadratic mean value of R_q_ = 4.14 μm and a maximum height of irregularities of R_z_ = 16.10 μm, which indicates a compact and stable film. After short-term annealing (5 min), a decrease in all roughness parameters (R_a_ = 0.614 μm, R_q_ = 0.781 μm and R_z_ = 3.09 μm) is observed, indicating temporary film compaction and leveling of minor irregularities. Prolonged heat treatment (15 min) leads to a sharp increase in roughness (R_a_ = 6.16 μm, R_q_ = 6.71 μm and R_z_ = 16.66 μm), reflecting the formation of large irregularities and porous areas on the surface. After 30 min, a moderate decrease in roughness is observed (R_a_ = 2.19 μm, R_q_ = 2.54 μm and R_z_ = 7.20 μm), while 45 min of annealing is accompanied by an increase in maximum irregularities (R_z_ = 20.20 μm) with average values of R_a_ = 2.88 μm and R_q_ = 3.53 μm. Prolonged annealing for 60 min again leads to surface leveling and a decrease in all roughness parameters (R_a_ = 2.20 μm, R_q_ = 2.34 μm and R_z_ = 6.43 μm). Thus, profile analysis shows that short-term annealing temporarily compacts the surface, reducing average roughness, while intermediate annealing times (15–45 min) lead to an increase in surface irregularities and porosity, and prolonged heat treatment (60 min) evens out the profile but retains some unevenness, which may affect the corrosion properties of the alloy. Electrochemical impedance spectroscopy and corrosion measurements of the amorphous alloy Al_87_Y_4_Gd_1_Ni_8_ in a 0.3% NaCl solution demonstrated a significant dependence of corrosion resistance on the duration of heat treatment. Figure 2 shows the evolution of the corrosion potential (E_corr_) and corrosion current density (*j*) as a function of annealing time. Figure 2 shows the dependence of the corrosion potential (E_corr_, black line, left axis) and corrosion current density (j, green line, right axis) of the amorphous alloy Al_87_Y_4_Gd_1_Ni_8_ in 0.3% NaCl on the heat treatment time. In the as-cast state, the alloy is characterized by a moderately negative corrosion potential and a relatively low corrosion current density, which corresponds to the formation of a dense and protective passive film. Short-term annealing (5 min) causes a shift of E_corr_ to a more negative side and a decrease in surface roughness, which indicates the compaction of the oxide film and a temporary increase in corrosion resistance. Further heat treatment (15–30 min) is accompanied by a shift of E_corr_ to a more negative region and a significant increase in current density. At the same time, the film resistance (R_f_) and charge transfer resistance (R_ct_) decrease sharply, and profile analysis records maximum roughness values (R_a_ ≈ 6.16 μm and R_z_ ≈ 16.66 μm at 15 min). This indicates the destruction of the passive layer, the formation of a porous structure and the development of local corrosion. Longer annealing (45–60 min) partially evens out the surface profile, but the potential remains more negative, and the corrosion current density remains high. This indicates that the oxide film remains defective and porous, which does not provide effective barrier protection. The analysis of E_corr_ and *j* data in combination with EIS and profilometry confirms that the highest corrosion resistance of the alloy is achieved in the initial state and after short-term annealing (5 min). Further increasing the heating time reduces the protective properties of the film due to its destruction and increased porosity.

### 4.2. Tribological Analysis

**Figure 3:**
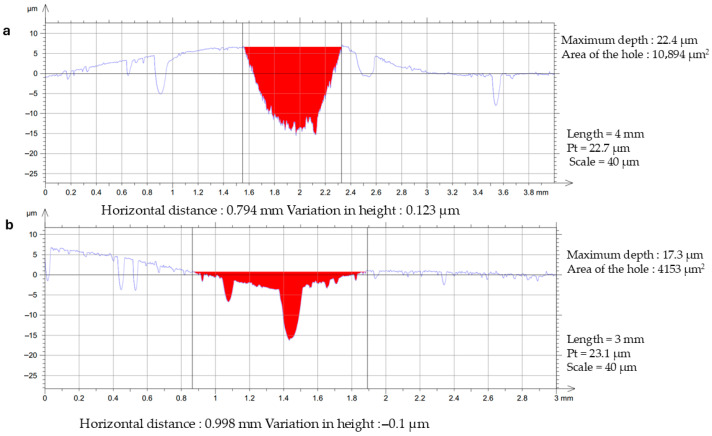
AMA surface profiles of the contact side (width × height) of Al_87_Y_4_Gd_1_Ni_8_ (**a**) initial and (**b**) annealed for 5 min at T_3_ = 611 ± 1 K after tribological tests.

After conducting tribological tests (Figure 3), the average surface area of wear marks was assessed using a Surftest SJ-500 contact profilometer.

The volumetric consumption parameter ($V_{w}$) after dry contact tests is 3.36·10^−3^ mm^3^/N·m for the initial AMA Al_87_Y_4_Gd_1_Ni_8_ and 3.78·10^−4^ mm^3^/N·m for AMA annealed for 5 min. The average friction coefficient (µ_a_) after dry contact tests is 2.355 and 0.628 for initial and annealed AMAs, respectively, which indicates that annealing for 5 min improves tribological properties.

### 4.3. XPS Analysis

The atomic concentration analysis reveals key trends in the evolution of surface composition. The Al/Y ratio decreases from 68.21 for the as-cast case to 25.93 for annealed AMA for 45 min, indicating progressive yttrium enrichment; however, a slight reversal of this trend is observed at 60 min of annealing (39.12).

The near-constant O/Al ratio, ranging between 1.4 and 1.6, strongly suggests the formation of a stable Al_2_O_3_ phase. Concurrently, the O/Y ratio shows a sharp decline from 109.21 for the as-cast case to 38.28 for AMA annealed for 45 min, consistent with yttrium segregation toward the surface. Nickel remains at trace concentrations below 0.1 at.% in all samples (Table 2). Carbon contamination decreases from 57.1 at.% for the as-cast sample to 37.66 at.% for AMA annealed for 30 min and then rises again at longer modification times, likely due to re-adsorption from the environment. These compositional changes reflect competing mechanisms, including yttrium surface diffusion, aluminum oxide passivation and dynamic carbon adsorption–desorption processes.

**Figure 4:**
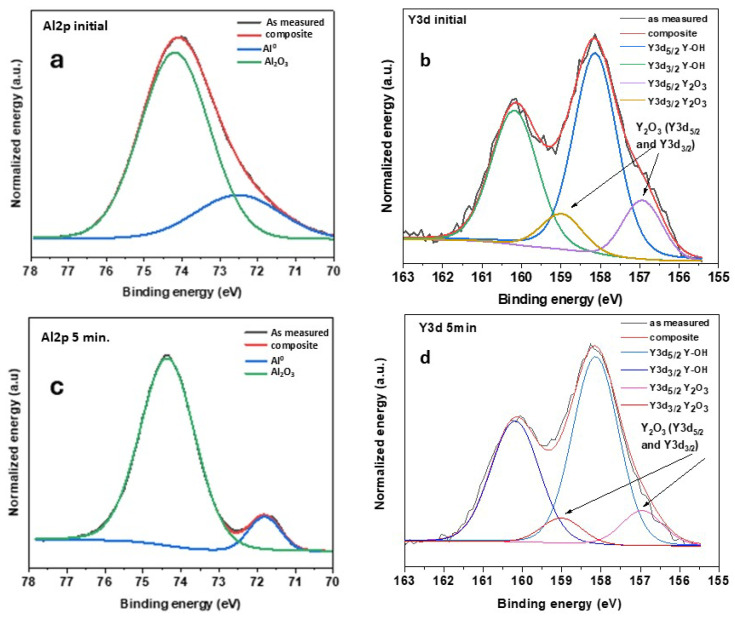
XPS spectra of (**a**,**c**) Al2p and (**b**,**d**) Y3d of AMAs Al_87_Y_4_Gd_1_Ni_8_, (**a**,**b**) initial and (**c**,**d**) annealed for 5 min.

High-resolution spectral analysis was performed for the Al2p and Y3d peaks. The gadolinium and nickel peaks were excluded from analysis due to their low surface atomic concentrations. Figure 4 presents the deconvolution results of the photoelectron emission lines: (a) Al 2p and (b) Y 3d.

The deconvoluted Al2p spectra (Figure 4) exhibited two components corresponding to different chemical environments. The metallic aluminum component (Al^0^) appeared at 71.3–71.8 eV with decreasing relative abundance from 14.9% (as-cast sample) to 5.3% (annealed for 60 min). This relatively broad range of binding energies is lower than the standard value for pure aluminum (72.6 eV) [48], though comparable values have been reported for both pure Al (71.5 eV) [49] and Al-Ni-Y alloys (72.0 eV) [48]. The oxide component (Al^3+^) at 74.2–74.4 eV dominated the spectra (85.2–94.7% of Al atoms are in this chemical state) and showed increasing predominance with modification time [50]. While the oxide structure remains relatively consistent—nearly constant O/Al atomic ratio across all samples—supporting the formation of a stable Al_2_O_3_ phase, the metallic aluminum environment varies significantly between samples, possibly reflecting local compositional fluctuations or differential surface segregation effects. The progressive decrease in the Al^0^ component and corresponding increase in the Al^3+^ component indicate the formation and growth of a passivating aluminum oxide layer.

The high-resolution Y 3d spectra (Figure 4, Table 3) consistently exhibited two distinct chemical states, each represented by spin-orbit doublets (Y 3d_5/2_ and Y 3d_3/2_ components separated by 2.05 eV). The lower binding energy state (peaks 1 + 3), with Y3d_5/2_ binding energies in the range of 156.8–157.3 eV, corresponds to Y_2_O_3_ or sub-stoichiometric Y_2_O_3-δ_ [51,52,53]. The second chemical state (peaks 2 + 4) appeared at higher binding energies (Y 3d_5/2_ in the range of 158.0–158.1 eV) and is attributed to hydroxylated yttrium species (Y–OH) [52,53]. The relative abundance of these two states changed systematically with the modification time; the contribution of Y_2_O_3_ decreased from 19.4% for the as-cast sample to 1.8% for AMA annealed for 60 min, while the Y–OH component increased from 80.6% to 98.2%, indicating progressive surface hydroxylation, likely due to exposure to ambient moisture. The O/Y ratio trend, calculated from atomic concentration, further supports yttrium segregation to the surface followed by interaction with hydroxylating species. A temporary increase in the Y_2_O_3_ fraction (17.3% compared to 14.9% at 5 min) suggests partial surface reoxidation during processing.

Overall, XPS analysis identified stable aluminum oxide passivation and progressive yttrium hydroxylation as dominant features of Al_87_Y_4_Gd_1_Ni_8_ surface chemistry, with kinetics controlled by competing segregation and oxidation.

### 4.4. Electrochemical Analysis

**Figure 5:**
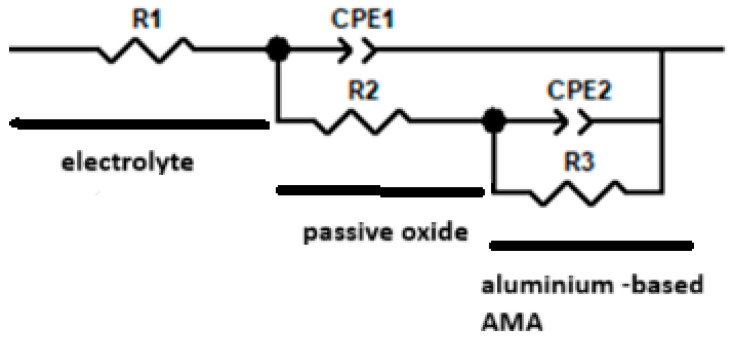
Equivalent electrical circuit used for fitting the EIS data of the amorphous Al_87_Y_4_Gd_1_Ni_8_ alloy in 0.3% NaCl solution.

**Figure 6:**
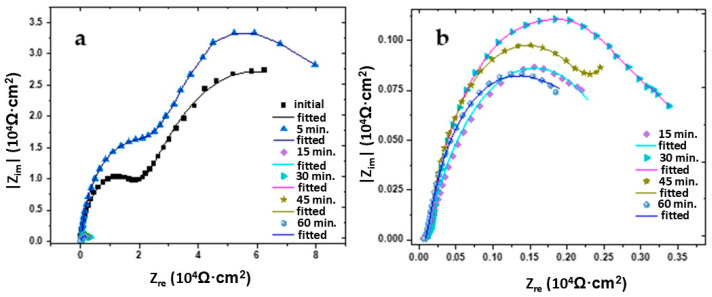
Nyquist plots of the amorphous Al_87_Y_4_Gd_1_Ni_8_ alloy in 0.3% NaCl (**a**) initial and at 5 min and (**b**) after different heat treatment conditions. Experimental points (appropriate shapes) and fits (lines) using the equivalent circuit from Figure 5 are shown.

**Figure 7:**
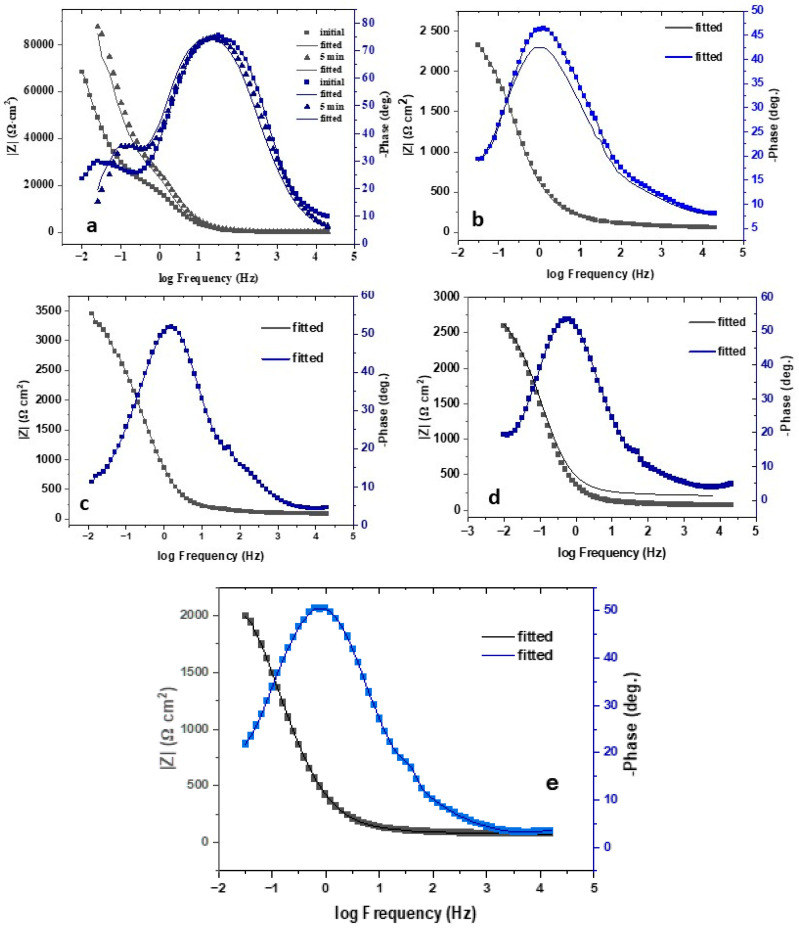
Bode plots (impedance magnitude and phase angle) of the amorphous Al_87_Y_4_Gd_1_Ni_8_ alloy in 0.3% NaCl (**a**) initial and at 5 min and (**b**–**e**) after different heat treatment conditions, with experimental data and fits from the equivalent circuit shown in Figure 5.

The diagram used to determine the impedance characteristics of the AMAs in 0.3% NaCl is shown in Figure 5. It includes the ohmic resistance of the electrolyte (Rs), the passive film characterized by the resistance of the oxide passive film R_f_ and CPE_f_, a constant phase element used to describe the non-ideal capacitance of the film on the electrode. Aluminum amorphous alloys in neutral NaCl form a passive oxide film: it is characterized by diffusion/ionic conductivity resistance through the film (R_f_) and non-ideal capacitance due to roughness/gradation of the composition (CPE_f_). At the metal–electrolyte interface, electron ion transfer (corrosion/anodic processes) occurs, which is determined by Rct, and the double layer behaves not as an ideal capacitor but as an ‘imperfect’ capacitance (CPE_dl_), which is typical for real rough and heterogeneous surfaces. The AMA–0.3% NaCl boundary describes the resistance to charge transfer across the phase boundary (R_ct_) and its CPEdl. According to this scheme, the approximation of the Nyquist and Bode data shown in Figure 6 and Figure 7, respectively, was performed.

Electrochemical impedance spectroscopy (EIS) and profile analysis of the surface of an amorphous alloy Al_87_Y_4_Gd_1_Ni_8_ in a 0.3% NaCl solution demonstrated significant changes in the topography and protective properties depending on the heat treatment time (Table 4). The system was modeled by an equivalent circuit consisting of a series-connected ohmic resistance of the solution (Rs) and two parallel circuits: the resistance and pseudocapacitance of the film (Rf∥CPEf) and the charge transfer resistance with a double electric layer (Rct∥CPEdl). The CPE (Q) parameter describes the pseudocapacitive properties of the surface; a value of n < 1 indicates roughness and heterogeneity of the film, while n ≈ 1 corresponds to an almost ideal capacitor.

Impedance characteristics were measured at open-circuit potential (OCP), and they are −630, −614, −707, −637, 606, 631 mV for the as-cast sample and according to the annealing times of 5, 15, 30, 45 and 60 min, respectively, at a temperature of 611 ± 2 K.

Electrochemical impedance spectroscopy (EIS) of an amorphous alloy Al_87_Y_4_Gd_1_Ni_8_ in a 0.3% NaCl solution showed significant changes in surface characteristics depending on the heat treatment time. Calculation of roughness through the effective double-layer capacitance shows that in the initial state (as cast), the surface is characterized by high film resistance (R_f_ ≈ 2.36·10^4^ Ω·cm^2^) and charge transfer resistance (R_ct_ ≈ 7.0·10^4^ Ω·cm^2^), and the roughness is 2.2, which corresponds to a compact but slightly rough surface. Short heat treatment for 5 min leads to temporary film densification and an almost perfect double layer (n_dl_ = 0.99), while the roughness increases to 7.5, reflecting the increased active contact area with the electrolyte. Prolonged treatment (15 min) causes a sharp decrease in film resistance and charge transfer to 80 Ω·cm^2^ and 2.9·10^3^ Ω·cm^2^, respectively (Table 4), indicating film disruption and increased corrosion activity; at the same time, an increase in Q and a decrease in nf are observed, reflecting high surface heterogeneity, while roughness decreases to 0.26. With further heat treatment (30–60 min), the film resistance remains low, and the charge transfer resistance is consistently low (~2.5–3.5·10^3^ Ω·cm^2^), indicating a decrease in the protective properties of the passivating layers; CPE values show high Q and n variations from 0.62 to 1.0, indicating the formation of a thin, porous, but capacitive film, while roughness ranges from 0.16 to 3.25, reflecting surface heterogeneity after prolonged annealing.

EIS shows that short heat treatment temporarily increases roughness and densifies the film, while longer annealing leads to film destruction, reduced resistance and roughness and a corresponding increase in the corrosion activity of the alloy.

In its as-cast state, the surface is characterized by high film resistance (R_f_ ≈ 2.36·10^4^ Ω·cm^2^) and charge transfer resistance (R_ct_ ≈ 7.0·10^4^ Ω·cm^2^), and roughness parameters are R_a_ = 2.83 μm, R_q_ = 4.14 μm and R_z_ = 16.10 μm, which corresponds to a compact, relatively homogeneous surface. Short-term annealing (5 min) causes a decrease in average roughness (R_a_ = 0.614 μm, R_q_ = 0.781 μm and R_z_ = 3.09 μm), a simultaneous increase in film resistance (R_f_ ≈ 3.89·10^4^ Ω·cm^2^) and almost ideal double-layer parameters (n_dl_ = 0.99), indicating temporary compaction and surface leveling. Longer annealing (15–30 min) leads to a significant increase in roughness (R_a_ = 6.16 μm, R_q_ = 6.71 μm and R_z_ = 16.66 μm at 15 min), the formation of large irregularities and porous areas and a simultaneous decrease in film resistance and R_ct_, reflecting partial destruction of the protective layer and increased corrosion activity. At 30–45 min, roughness fluctuates (R_a_ = 2.19–2.88 μm, R_q_ = 2.54–3.53 μm and R_z_ = 7.20–20.20 μm), while resistances remain low, indicating a porous, less protective film. Prolonged heat treatment of 60 min evens out the profile (R_a_ = 2.20 μm, R_q_ = 2.34 μm and R_z_ = 6.43 μm), but CPE shows high Q values and variable n (0.62–1.0), reflecting a thin, porous and heterogeneous surface.

As can be seen in Figure 2, in its initial state, the AMAs has the lowest negative E_corr_ value. After short-term annealing (5 min), the potential shifts to a more negative side, indicating a decrease in corrosion resistance. A further increase in heating time (15–30 min) causes E_corr_ to shift to a more negative side, which means a decrease in durability and activation of corrosion processes. During prolonged heat treatment (45–60 min), the potential remains at a negative level, slightly leveling out, but the protective properties remain weakened. The value of the corrosion current density (*j*) with heat treatment time has a clear tendency to increase. The lowest values are recorded for the initial state and short annealing (5 min), while at 15–60 min, j increases significantly, indicating an intensification of anodic dissolution processes. The Figure 2 shows that short annealing (≈5 min) is the optimal mode, providing minimum *j* values. On the other hand, prolonged annealing (15–60 min) leads to a shift of the potential to a more negative region and an increase in the corrosion current, which means a significant decrease in corrosion resistance.

The results of EIS and profile analysis indicate complex interactions between the surface structure, chemical composition of the alloy and aggressive environment. The amorphous alloy Al_87_Y_4_Gd_1_Ni_8_ is characterized by high metastability and uneven distribution of elements. Aluminum, as the main component, actively oxidizes, forming an oxide protective layer, while rare-earth elements Y and Gd are capable of increasing the stability of the film and forming local protective phases. Nickel, which is present in the composition, contributes to increasing the electrical conductivity and corrosion resistance of the film by forming a denser passive phase.

The introduction of an amorphous structure ensures the absence of classic grain boundaries, which usually serve as active corrosion sites, but the heterogeneity of the distribution of elements and the roughness of the surface form local active centers. Under the action of chlorine ions (Cl^−^), aggressive erosion of the oxide layer is observed, which is especially noticeable during intermediate and prolonged heat treatment, when the surface roughness increases and the film becomes porous. Chloride ions are able to penetrate micropores, initiating local corrosion and reducing charge transfer resistance.

In addition to Cl^-^ ions, the following factors influence surface formation:

1. Microstructural effects: Local concentrations of Y and Gd can increase or decrease film stability.
2. Thermal diffusion: During annealing, elements are displaced, changing the roughness and porosity of the film.
3. Mechanical stresses: Stresses formed during crystallization or heat treatment can contribute to microcracks in the film.
4. Chemical heterogeneity of the oxide film: The presence of various oxides (Al_2_O_3_ and Y_2_O_3_) forms zonal differences in protective properties.

The observed changes in roughness, film resistance and CPE parameters can be explained both by the interaction of the alloy composition and amorphous structure and by the aggressive action of chloride ions, which initiate local corrosion centers. Short-term annealing allows the film to be temporarily compacted and ion permeability to be reduced, while intermediate and long-term annealing increase porosity and roughness, making the surface more susceptible to chloride corrosion.

During heat treatment, atoms of Y, Gd and Ni diffuse into the upper layers of the alloy, changing the local composition of the film and its roughness. Short-term annealing (5 min) leads to film densification, defect reduction and a temporary decrease in chlorine ion permeability, which manifests itself in a decrease in roughness parameters and an increase in film resistance (R_f_). Intermediate annealing times (15–30 min) cause an increase in roughness and the formation of local porous areas through which Cl^-^ ions can easily penetrate, initiating local corrosion centers, which is reflected in a sharp decrease in R_ct_ and an increase in Q in CPE. Prolonged annealing (≥45 min) partially evens out the surface, but porosity and defects remain, which supports the diffusion of chloride ions and limits the formation of a stable protective film.

The corrosion activity of the alloy is determined not only by electrochemical parameters but also by the electronic structure of the component atoms, which affects the formation of oxide layers, as well as structural defects, roughness and diffusion of Cl^-^ ions into micropores. Other potential factors include local stresses in the film, chemical heterogeneity of oxides and interactions between different oxides (Al_2_O_3_ and Y_2_O_3_), which create zonal differences in protective properties. These results highlight the need to optimize the time and conditions of heat treatment to achieve maximum film densification and minimize the diffusion of aggressive ions, ensuring effective protection of amorphous Al alloys against corrosion.

The amorphous structure of the alloy eliminates the classic grain boundaries, which usually serve as active corrosion centers, but surface roughness and local chemical composition heterogeneity create micropores, defects and irregularities through which active ions, in particular chloride ions, can diffuse. Due to its small ionic radius and high electron density, Cl^−^ easily penetrates pores and microcracks, interacting with the oxide film and locally cleaving Al^3+^, which initiates the formation of active corrosion centers. At the atomic level, this corresponds to the local destruction of Al–O bonds and the diffusion of ions through defective areas of the film. During heat treatment, Y, Gd and Ni atoms diffuse into the upper layers of the alloy. Short-term annealing (5 min) promotes film densification, defect reduction and surface leveling, which limits Cl^−^ penetration and increases charge transfer resistance. Intermediate annealing times (15–30 min) form local porous areas and increase roughness, which increases the diffusion of chloride ions into defects and promotes local depassivation. In this case, the CPE parameters reflect the increased heterogeneity of the double layer and film, and the decrease in R_ct_ indicates active corrosion centers. Prolonged annealing (≥45 min) partially evens out the surface profile, but the porous areas remain, maintaining Cl^−^ diffusion and reducing the stability of the protective layer.

Aluminum atoms with three valence electrons in the outer p-shell oxidize rapidly, forming a dense and passivating layer of Al_2_O_3_, which blocks further electron migration and limits corrosion. Rare-earth elements Y (electron configuration [Kr]4d^1^5s^2^) and Gd ([Xe]4f^7^5d^1^6s^2^) in the alloy are capable of forming oxide inclusions (Y_2_O_3_ and Gd_2_O_3_) with high local electron density, which stabilizes the film and increases its mechanical strength. Nickel with filled d-orbitals promotes the formation of dense NiO impurities, increasing the film’s electrical conductivity and its resistance to electrolyte penetration.

A key factor is the electronic interaction between alloy atoms and chlorine ions: Al^3+^, Y^3+^ and Gd^3+^ in the film locally change the distribution of electron density, creating fields that direct the movement of Cl^−^ into defects, while NiO inclusions increase local electron conductivity and promote electrochemical reactivity. This mechanism explains why short-term annealing temporarily reduces ion diffusion, while intermediate and long-term annealing increase active corrosion sites.

## 5. Conclusions

1. Profile analysis of initial and annealed AMAs showed that roughness parameters (Ra, Rq and Rz) fluctuate significantly depending on the annealing duration. The lowest values were observed after short-term annealing (5 min), while further increases in time led to the formation of more pronounced surface micro-relief.
2. Five minute isothermal annealing at T_3_ = 611 ± 2 K improves tribological properties due to surface development and the formation of oxide layers on the AMA surface.
3. Aluminum and nickel form protective oxide films, while the presence of rare-earth elements (Y and Gd) contributes to the stabilization of the structure but does not always prevent the penetration of aggressive ions.
4. The Cl^−^ ions are actively adsorbed on the surface and penetrate microcracks and defects, causing local destruction of oxide films. This leads to an increase in roughness during certain annealing periods, which is particularly evident in the fluctuations of the R_z_ parameter.
5. The EIS method has established that short-term heat treatment temporarily increases roughness and compacts the film, while longer annealing leads to film destruction, reduced resistance and roughness and a corresponding increase in the corrosion activity of the alloy.

## Figures and Tables

**Table 1:** Surface roughness parameters (R_a_, R_q_ and R_z_) of the amorphous Al_87_Y_4_Gd_1_Ni_8_ alloy after different annealing times.

Roughness, µm	Time of Heat Treatment, min					
	As Cast	5	15	30	45	60
R_a_	2.83	0.61	6.16	2.19	2.88	2.20
R_q_	4.14	0.78	6.71	2.54	3.53	2.34
R_z_	16.10	3.09	16.66	7.20	20.20	6.43

**Table 2:** Atomic concentrations and relative ratios for selected elements.

AMA, Time of Annealing, Min	Atomic Concentration					Relative Ratio of Atomic Concentration		
	Al	Ni	Y	O	C	O/Al	O/Y	Al/Y
initial	16.37	0.07	0.24	26.21	57.10	1.60	109.21	68.21
5	21.55	0.07	0.40	30.57	47.41	1.42	76.43	53.88
15	20.20	0.03	0.38	28.49	50.90	1.41	74.97	53.16
30	25.65	0.05	0.66	35.98	37.66	1.40	54.52	38.86
45	18.41	0.03	0.71	27.18	53.67	1.48	38.28	25.93
60	19.17	0.04	0.49	27.66	52.63	1.44	56.45	39.12

**Table 3:** Quantitative evolution of Y chemical states.

Modification Time, min	Y_2_O_3_ Component	Y-OH Component
Initial	19.4%	80.6%
5	14.5%	85.5%
15	17.3%	82.7%
30	12.5%	87.5%
45	8.9%	91.1%
60	1.8%	98.2%

**Table 4:** Impedance parameters of the amorphous Al_87_Y_4_Gd_1_Ni_8_ alloy in 0.3% NaCl solution obtained from equivalent circuit fitting (Figure 5) after different annealing times.

Time of Annealing, min	R_1_, Ω·cm^2^	R_2_, Ω·cm^2^	CPE-f-T (Qf)·10^−5^, Ω^−1^cm^−2^	n_f	R_3_ Ω·cm^2^	CPE-dl-T (Qdl)·10^−5^, Ω^−1^cm^−2^	n_dl	Roughness (Rf/Qdl), ·10^3^
as cast	74.8	23,585	0.67	0.89	70,056	10.6	0.80	2.23
5	130.9	38,914	0.61	0.88	57,862	5.2	0.99	7.51
15	58.1	80.1	14.0	0.59	2896	30.6	0.72	0.26
30	91.3	207.5	25.0	0.62	3506	6.4	1.00	3.25
45	71.3	68.1	40.1	0.65	2976	34.2	0.88	0.20
60	74.5	50.1	33.0	0.67	2510	31.0	0.82	0.16

## Data Availability

The original contributions presented in this study are included in the article. Further inquiries can be directed to the corresponding author.

## Abbreviations

The following abbreviations are used in this manuscript:

AMA	Amorphous metal alloy
OCP	Open circuit potential
SCE	Saturated calomel electrode
XPS	X-ray photoelectron spectroscopy
EIS	Electrochemical impedance spectroscopy
